# Discrepancies in endpoints between clinical trial protocols and clinical trial registration in randomized trials in oncology

**DOI:** 10.1186/s12874-018-0627-2

**Published:** 2018-12-12

**Authors:** Victoria J. Serpas, Kanwal P. Raghav, Daniel M. Halperin, James Yao, Michael J. Overman

**Affiliations:** 1 Internal Medicine Residency Program, Baylor College of Medicine/ The University of Texas MD Anderson Cancer Center, 1 Baylor Plaza, Houston, TX 77030 USA; 2 Department of Gastrointestinal Medical Oncology, The University of Texas MD Anderson, 1515 Holcombe Blvd, Unit 426, Houston, TX 77030 USA

**Keywords:** Registry, Endpoints, Reporting

## Abstract

**Background:**

Clinical trials are an essential part of evidence-based medicine. Hence, to ensure transparency and accountability in these clinical trials, policies for registration have been framed with emphasis on mandatory submission of trial elements, specifically outcome measures. As these efforts evolve further, we sought to evaluate the current status of endpoint reporting in clinical trial registries.

**Methods:**

We reviewed 71 oncology related randomized controlled trials published in three high impact journals. We compared primary (PEP) and non-primary endpoints (NPEP) between the clinical trial protocols of these trials and their corresponding registration in one of the 14 primary global clinical trial registries. A discrepancy was defined as the non-reporting or absence of an endpoint in either the protocol or registry. The primary endpoint was the rate of discrepancy between secondary endpoints in clinical trial protocols and clinical trial registries.

**Results:**

Of the 71 clinical trials, a discrepancy in PEP was found in only 4 trials (6%). Secondary endpoint (SEP) differences were found in 45 (63%) trials. Among these 45 trials, 36 (80%) had SEPs that were planned in the protocol but not reported in the registry and 19 (42%) had SEPs with endpoints in the registry that were not found in the protocol. The total number of SEPs that were absent from the corresponding registry and protocol were 84 and 29, respectively. Of these endpoints, 48 (57%) and 9 (31%) were included in the published report of these trials.

**Conclusion:**

Although recent regulations and enhanced procedures have improved the number and quality of clinical trial registrations, inconsistencies regarding endpoint reporting still exist. Though further guidelines for the registration of clinical trials will help, greater efforts to provide a correct, easily accessible, and complete representation of planned endpoints are needed.

**Electronic supplementary material:**

The online version of this article (10.1186/s12874-018-0627-2) contains supplementary material, which is available to authorized users.

## Background

Randomized controlled trials (RCT) are the cornerstone of evidence-based medicine. Due to their important effect on clinical practice, there is a necessity to have transparent reporting of RCT results. However, despite the inherent ethical obligation and benefit of non-biased clinical reporting, there remains a noticeable evidence of selective reporting in clinical trials. Selective reporting can include under-reporting, over-reporting, or misreporting. For this reason, mandates to register clinical trials in publically accessible databases were enacted. Providing information in a public database not only facilitates research being done in a more transparent way, but may also enhance recruitment into clinical trials and avoid unnecessary duplication of research.

The first U.S. federal law to require clinical trial registration was the US Food and Drug Administration (FDA) Modernization Act of 1997 [1]. Following this, in 2000, the public access online clinical trial registry, clinicaltrials.gov, was created by the National Institutes of Health (NIH) [2]. In the years following, many other groups including the International Committee of Medical Journal Editors (ICMJE), the World Health Organization (WHO), and the World Medical Association created similar mandates requiring registration of clinical trials in a public registry [3–8]. In 2016, the department of Health and Human Services delivered a final rule dictating that all NIH- funded clinical trials be registered regardless of whether they are covered by the FDA requirements [9, 10]. Through these mandates, clinical trial registration has grown and become the norm.

Recently, there have been even further efforts by individual journals to improve clinical trial transparency by requiring the provision of the clinical trial protocol for randomized controlled trials [11]. We have previously found that when comparing protocols to final publications, there is a high rate of non-reporting and unplanned endpoint reporting [12]. As the protocol is the guiding resource for clinical trial registration, we sought to evaluate the ability of public registries to accurately represent the endpoints from oncology related randomized controlled trial protocols. Discrepancies between protocol and manuscript were previously reported using the same data set [12]. As primary endpoint discrepancies have been previously well studied, we choose to focus on secondary endpoint reporting.

## Methods

### Clinical trial and registry identification

As previously reported, a PubMed search was performed on December 1, 2013 for the key words randomized, randomly, or random in either the title or the abstract [12]. Results were filtered with the subject of cancer and were limited to articles published in *Journal of Clinical Oncology* (*JCO*), the *New England Journal of Medicine* (*NEJM*), or *The Lancet* between March 1, 2012, and December 31, 2012. These journals were chosen based on their impact factor (> 15) and the availability of the corresponding protocols as required for randomized trials by these journals.

Within the WHO Registry Network, there are currently 14 primary registries that meet the requirements of the ICMJE [13]. These registries were searched, using trial title and identification number, and 71 of the 74 trials were located. ClinicalTrials.gov was the preferred registry if trials were included in more than one registry. The four trials without identified registration were excluded. The last date of examination of the online registries was September 1, 2016. Protocols were available for all clinical trials.

### Evaluation of clinical trial registries and protocols

For each randomized trial, the protocol, published report, and registry were reviewed and compared for objectives, endpoints, and statistical analysis plan. Both primary and non-primary endpoints, including secondary and exploratory, were compared. Endpoints were determined from the protocol with preference given to sections in this order: end points, objectives, and statistical plans. Exploratory endpoints were those included in endpoint sections labeled as “exploratory”, “translational”, “correlative”, and “tertiary.” When comparing endpoints from protocol and registry, a discrepancy was defined as the non-reporting or absence of an endpoint in either the protocol or registry when listed in the other. Endpoint reporting discrepancies were grouped into two major categories: either absent endpoints in the registry in comparison to the protocol or extra endpoints in the registry not in the protocol. Endpoints that were either downgraded from primary in protocol to secondary in registry or upgraded from secondary in protocol to primary in registry were also noted. Endpoints from manuscripts, protocols, and registries were evaluated by two authors, with a third author reviewing any inconsistent findings. Determination of endpoint discrepancy was based upon intent, and thus a difference in wording was not incorporated as a discrepancy. For example, “safety”, “toxicity”, and “adverse events” were all considered the same endpoint. In addition to collecting endpoints from the registry, the clinical trial archives were searched for changes made to registry endpoints over time, though archives were only available for ClinicalTrials.gov.

In addition to endpoints, information was also collected regarding trial phase (II, III, or unspecified), tumor type, intervention type (systemic or non-systemic), trial initiation period (before or after 2005), trial enrollment date (before or after registration), trial outcome (positive or negative), funding source (pharmaceutical or non-pharmaceutical), number of endpoints (more or less than median), duration of trial (more or less than median), and protocol length (more or less than median). Trial outcome was considered positive if the study reported that it met the PEP and considered negative if the study failed to meet the PEP.

### Statistical methods

The objective of this study was to determine the consistency of endpoint reporting between protocol and clinical trial registry. The primary endpoint was the rate of discrepancy between secondary endpoints in clinical trial protocols and clinical trial registries. Statistical significance between categorial variables was determined by Fisher’s exact test and a two-sided *p*-value of ≤0.05 was considered statistically significant.

## Results

### Trial characteristics

We identified a total of 71 oncology-based randomized trials published in the Journal of Clinical Oncology (*n* = 52), New England Journal of Medicine (*n* = 17), and Lancet (*n* = 2), Table 1. A majority of the trials were Phase III (*n* = 57), were initiated between 2006 and 2010 (*n* = 41), were funded by pharmaceutical companies (*n* = 38), and involved systemic therapy as the intervention (*n* = 48). The trials were registered in ClinicalTrials.gov (*n* = 62), the International Standard Randomized Controlled Trial Number registry (n = 5), the European Union Drug Regulating Authorities Clinical Trial Registry (*n* = 2), the Australia New Zealand Clinical Trials Registry (*n* = 1), and the Netherlands National Trial Register (*n* = 1).

**Table 1:** Baseline clinical trial characteristics

	Variables	Number of trials	(%)
Phase	II	10	(14)
	III	57	(80)
	Unspecified	4	(6)
Type of Tumor	Breast	13	(18)
	Genitourinary	10	(14)
	Gastrointestinal	6	(8)
	Lung	10	(14)
	Other solid tumors	14	(20)
	Hematological	12	(17)
	Any cancer	6	(8)
Publication	JCO	52	(73)
	NEJM	17	(24)
	Lancet	2	(3)
Registry	Clinicaltrials.gov	62	(87)
	ISRCT	5	(7)
	EudraCT	2	(3)
	ANZCTR	1	(1)
	NTR	1	(1)
Type of Intervention	Chemotherapy	22	(31)
	Targeted therapy	26	(37)
	Chemotherapy and radiation	6	(8)
	Surgery or radiation	6	(8)
	Supportive care	11	(15)
Trial Initiation Period	1994–2005	30	(42)
	2006–2010	41	(58)
Trial Result	Negative	37	(52)
	Positive	34	(48)
Funding Source	Non-pharma	33	(46)
	Pharma	38	(54)
Length of Trial	< 3 years	30	(42)
	≥ 3 years	41	(58)
Protocols with endpoints	Primary endpoints	71	(100)
	Secondary endpoints	71	(100)
	Exploratory endpoints	28	(39)

*Abbreviations: JCO* Journal of Clinical Oncology, *NEJM* New England Journal of Medicine, *ISRCT* International Standard Randomized Control Trial, *EudraCT* European Clinical Trials Database, *ANZCTR* Australian New Zealand Clinical Trial Registry, *NTR* Netherlands National Trial Register

### Primary endpoint discrepancies

Out of the 71 trials, only 4 trials had a discrepancy in primary endpoint reporting between the protocol and registry (6%), Table 2. Of these, 3 had multiple PEP listed in the protocol but only one PEP in the registry, with the other endpoints included as SEP in the registry. The remaining trial had the PEP of progression-free survival (PFS) in the protocol but listed time-to-progression (TTP) as the PEP in the registry. An additional file is included which contains a listing of all endpoints (Additional file 1).

**Table 2:** Reporting of clinical trial endpoints between clinical trial protocols and clinical trial registries

Endpoint Reporting	Trials	(%)	Endpoints	(%)
Clinical trial protocols with primary endpoints	71		75	
Primary Endpoint Discrepancies	4	(6)	4	(5)
Clinical trial protocols with secondary endpoints	71		507	
Secondary Endpoint Discrepancies	45	(63)	113	(22)
Secondary endpoints absent from registry	36	(51)	84	(17)
Included in published report	27	(38)	48	(9)
Secondary endpoints absent from protocol	19	(27)	29	(6)
Included in published report	7	(10)	9	(2)
Present in non-endpoint section of protocol	11	(15)	14	(3)
Clinical trial protocols with exploratory endpoints	28		96	
Exploratory endpoints absent from registry	25	(89)	89	(93)

### Non-primary endpoint discrepancies

Discrepancy between registry and protocol for secondary endpoints was seen in 45 trials (61%), Table 3. Exploratory endpoints were present in 28 protocols but were reported in the registries in only 3 trials. In part this reflects both clinical trial registries construction and guidance, in which no exploratory endpoint section exists for endpoint reporting and no registries require reporting beyond primary and secondary endpoints. Due to the limited reporting of exploratory endpoints, these were not further evaluated.

**Table 3:** Secondary endpoint discrepancies

	Number of trials *n* = 71 (%)	Secondary Endpoint Discrepancy *n* = 45 (%)	*P* Value
Trial Initiation Period			
1994–2005	30 (42)	19 (42)	0.9944
2006–2010	41 (58)	26 (58)	
Trial Result			
Negative	37 (52)	26 (58)	0.2087
Positive	34 (49)	19 (42)	
Funding Source			
Non-pharma	33 (46)	18 (40)	0.1499
Pharma	38 (54)	27 (60)	
Duration of Trial			
< 3 years	30 (42)	21 (47)	0.3220
≥ 3 years	41 (58)	24 (53)	
Number of clinical trial protocol non-primary endpoints^a^			
≤ 6	36 (51)	19 (42)	0.0600
> 6	35 (49)	26 (58)	
Protocol Length^a^			
≤ 34 pages	36 (51)	22 (49)	0.4969
> 34 pages	35 (49)	23 (51)	
Study Phase^b^			
II	10 (14)	7 (16)	0.8984
III	57 (50)	35 (78)	
Study Intervention			
Systemic	53 (75)	37 (82)	0.0536
Non-systemic	18 (25)	8 (18)	
Protocol Type			
Full	60 (85)	39 (87)	0.5162
Appended	11 (15)	6 (13)	

^a^Dichotomized at the median

^b^4 unspecified trials not included

Secondary endpoint discrepancies did not differ with regard to trial initiation period, duration of trial, or outcome of trial. Of note, there was also no statistical difference in discrepancy rate in those with enrollment prior to registration and those with registration prior to enrollment (*p* = 1.00). Though not statistically significant, SEP discrepancies were more commonly seen in industry sponsored compared to non-industry sponsored trials (71% vs. 55%, *p* = 0.149, odds ratio 2.05, 95% CI: [0.77, 5.42]), in trials involving systemic interventions vs non-systemic interventions (70% vs 44%, *p* = 0.054, odds ratio 0.35, 95% CI: [0.12, 1.02]), and in clinical trials with > 6 more compared to < 6 planned NPEP (74% vs. 53%, *p* = 0.060, odds ratio 2.58, 95% CI: [0.96, 6.98]).

### Secondary endpoints absent from registry

In 36 of the 45 trials with SEP discrepancies, there were endpoints reported in the protocol that were not included in the registry, Table 4. Twenty-seven of these trials had discrepant endpoints that were included in the final published report, with 19 of those being included in the report abstract.

**Table 4:** Secondary endpoint discrepancies stratified by endpoints absent from the protocol and absent from the registry

	Number of trials *n* = 71 (%)	Secondary Endpoints absent from registry *n* = 36 (%)	*P* value	Secondary Endpoints absent from protocol *n* = 19 (%)	*P* value
Trial Initiation Period					
1994–2005	30 (42)	15 (42)	0.9191	8 (42)	0.9878
2006–2010	41 (58)	21 (58)		11 (58)	
Trial Result					
Negative	37 (52)	22 (61)	0.1237	10 (53)	0.9578
Positive	34 (49)	14 (39)		9 (47)	
Funding Source					
Non-pharma	33 (46)	13 (36)	0.0757	8 (42)	0.6551
Pharma	38 (54)	23 (64)		11 (58)	
Duration of Trial					
< 3 years	30 (42)	18 (50)	0.1802	9 (47)	0.5979
≥ 3 years	41 (58)	18 (50)		10 (53)	
Number of protocol non-primary endpoints^a^					
≤ 6	36 (51)	15 (42)	0.1224	9 (47)	0.7340
> 6	35 (49)	21 (58)		10 (53)	
Protocol Length^a^					
≤ 34 pages	36 (51)	18 (50)	0.8292	8 (42)	0.0326
> 34 pages	35 (49)	18 (50)		11 (58)	
Study Phase^b^					
II	10 (14)	6 (17)	0.5993	3 (16)	0.3972
III	57 (50)	29 (81)		14 (74)	
Study Intervention					
Systemic	53 (75)	30 (83)	0.0880	15 (79)	0.7624
Non-systemic	18 (25)	6 (17)		4 (21)	
Protocol Type					
Full	60 (85)	31 (86)	0.7048	18 (95)	0.2670
Appended	11 (15)	5 (14)		1 (5)	

^a^Dichotomized at the median

^b^4 unspecified trials not included

**Fig. 1:**
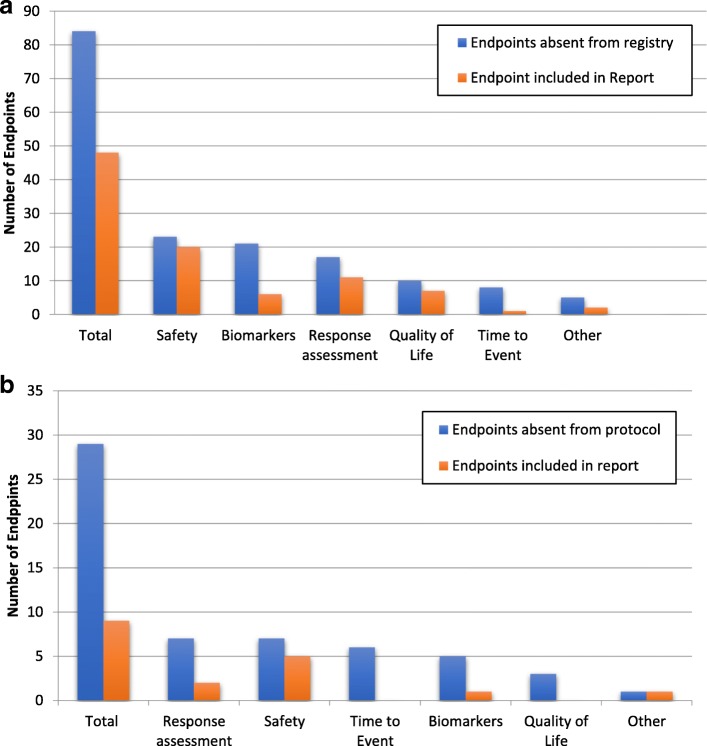
(**a**) Secondary endpoints absent in registry but present in protocol and (**b**) Secondary endpoints absent in protocol but present in registry

The total number of secondary endpoints among the 45 trials that were absent from the registry was 84 (median 2, range 1–8). The categorization of these endpoints is shown in Fig. 1a, with the most common categories being safety (27%), biomarkers (25%), or response assessment (20%). Of these endpoints, 48 were included in the report results with 22 of them also being included in the report abstract. 67 of these 84 endpoints were from trials registered at ClinicalTrials.gov. Interestingly, 53 of the 67 endpoints were not in any of the earlier versions of the registry (79%). To assess the importance of various endpoints for a clinical trial, we looked at those endpoints that were included in the final published report. This demonstrated that of the 14 that were in earlier versions of the registry but not included in the most current version, 10 of these were also included in the final published report.

### Secondary endpoints absent from protocol

In 19 of the 45 trials with SEP discrepancies, the registry included secondary endpoints not reported in the protocol, Table 4. Within those 19 trials, there were 29 secondary endpoints (median 1, range 1–5) included in the registry of which 9 were included in the published report. The categories of these endpoints were response assessment (24%), safety (24%), time to event (21%), biomarkers (17%), and quality of life (10%), Fig. 1b.

We assessed whether discrepancies were related to the variability regarding protocol construction and the information provided in the appended protocols. Of these 19 clinical trial protocols, a specific endpoint section was present in 3 and an objective section for those protocols without an endpoint section was present in 14. In the remaining 2 trials, no endpoint or objective section was present and endpoints were obtained from the statistics sections. Of the 29 SEPs included in the registry, 14 endpoints were reported in the protocol in other sections of the protocol such as “statistics”, “methods”, “measurements”, or “analysis”. 5 of these were also included in the published report. Of the 15 secondary endpoints not found in any section of the corresponding protocol, 4 were included in the published report.

## Discussion

Despite the ethical obligation and legal regulation of clinical trial reporting and registration, there remain discrepancies in endpoint reporting between protocol, published report, and registry. To our knowledge, this is one of the first efforts in oncology to evaluate the consistency of endpoints between the protocol and clinical trial registry. We found that a significant number of registered RCTs had secondary endpoints present in the protocol but absent in the registry (45 of 71, 63%). More critically, we found that over half of the missing endpoints were included in the published report (48 of 84, 54%). These findings demonstrate the limitations of using clinical trial registries in the assessment of endpoint reporting.

Surprisingly, there were also SEPs included in the registry that had not been listed in the protocol. In most clinical trial protocols, endpoints were listed in either the “endpoint” section or if an “endpoint” section was not present then the “objective” section. However, some endpoints that were included in the registry or report could be found in other sections of the protocol. By searching all sections of the individual protocols for missing endpoints we found endpoints in areas of the protocol such as “methods”, “measurements”, “statistics” and “analysis”. Based on this, it appears that this discrepancy is largely due to issues regarding protocol construction. In part, this may reflect the increasing complexity of protocols, wherein the mean and median length of the full or appended protocol was 44 and 34 (range 7–146), respectively. One limitation of this analysis was that no prior versions of the protocol were accessible and we were only able to evaluate the protocol or appended protocol provided with the published report.

Limited reporting of translational or exploratory endpoints reflects the current requirements for endpoint reporting within registries. The requirements for clinical trial reporting are determined by recommendations from the ICMJE, which designates minimum criteria required of each registered trial including that the registry must be accessible to the public, open to all prospective registrants, be managed by not-for-profit organization, have a method of ensuring validity, and be electronically searchable. In addition, the ICMJE and ClinicalTrials.gov both require minimum information to be registered for each clinical trial. Both organizations require a study hypothesis, definitions of the primary and secondary endpoints, and eligibility requirements among other information to be submitted for each registered trial [6]. For clinical trials registered in ClinicalTrials.gov, there is an option to include exploratory or translational endpoints in the registry as “other pre-specified outcomes”. However, none of the trials included in our study had designated endpoints in the “pre-specified outcomes” section of the registry. If these endpoints were registered, they were included as secondary endpoints. The limited reporting of exploratory endpoints in registries represents a critical problem with regard to the interpretation of unplanned endpoint reporting as the totality of endpoints are not known in the registry. We identified 28 of 71 studies with exploratory endpoints in the protocol and only 3 of these studies included these endpoints in the registry.

Recently, the Department of Health and Human Services has released a finalized rule regarding ClinicalTrials.gov with the aim to clarify the statutory language and expand transparency beyond the basic statutory requirements [9]. All US interventional trials studying a FDA-approved product other than Phase I trials are required to have registration and results information reporting. In regards to endpoint reporting, each trial is required to register primary endpoints as well as secondary endpoints which are defined as outcome measures that are “of lesser importance than a primary outcome measure but still included in the statistical analysis plan (SAP)”. The rule states that endpoints that are not in the SAP are not required for reporting. The regulation also requires the submission of a copy of the full protocol and SAP at the time of results information submission. We believe the provision of protocols and SAP is a beneficial step to transparency. However, based on our data, the discrepancies between protocol and registries will likely remain a persistent problem. The inaccuracy of registry endpoints is of great importance as the lengths of protocols generate difficulty with regard to ease of access for the end-user.

Multiple studies have shown that adherence to the former FDA regulation regarding ClinicalTrials.gov registration is poor, and despite previous guidelines and requirements trial registration has remained suboptimal [14, 15]. One study points out that although the FDA requires results reporting within 1 year of trial completion, only 13% of trials adhered to this and the average time of reports reporting was 17 months [16]. Though the impact of the most recent recommendations of ClinicalTrials.gov is not known at this time, there remains concern in regard to the completeness of adherence to these guidelines.

The selective reporting of clinical trial endpoints has been well demonstrated between published reports and clinical trial registration and between published reports and protocols [17–21]. For example, one study found discrepancies in endpoint reporting, cohort, intervention, or results between high impact journal publications and ClinicalTrials.gov were in as high as 97% of trials [17]. In addition, evidence of primary outcome discrepancies between publication and registry exist [21]. The focus of this report differs from prior publications as we sought to explore discrepancies between two so called gold standards for endpoints, the registry and the protocol. This is of particular interest as efforts increasing relying upon clinical trial registration need to ensure an accurate and complete representation exists in such registries. Our data suggest that the translation of clinical trial protocol endpoints into clinical trial registries is suboptimal and efforts to ensure alignment of endpoints between protocols and publications at the time of reporting are needed.

It is the authors’ belief that a fundamental reason for inaccuracies in endpoint reporting in part reflects the two issues of protocol construction and limited efforts to enforce alignment of planned endpoints at the time of trial publication. We have previously proposed a Comprehensive Outcomes Reporting in Randomized Evidence (CORRE) initiative to improve the outcome reporting in randomized trials [12]. The clear linkage of objectives to endpoints to statistical analysis proposed in CORRE should be incorporated into clinical trial protocol construction to improve the clarity of clinical trials. In addition, the CORRE initiative recommends a supplemental table at the time of publication to clearly align all reported endpoints and analyses from the protocol and the report. We strongly believe that efforts by journal editors to enforce clear and transparent reporting of endpoints is critical to provide an efficient and accurate assessment of the reported results for the end-user.

This study likely underestimates the full extent of trial reporting discrepancies as it was focused on the specific question regarding endpoint reporting. A limitation of this study was that clinical trial registries are updated throughout the lifespan of a trial. This means that registered endpoints may have been changed throughout that time. In the case of ClinicalTrials.gov, registry archives are available which allowed us to search the archives for changes within the registry. However, it was the only registry that had this option. Another limitation of the study was that it included clinical trials initiated before and after the FDA Modernization Act of 1997 and the creation of ClinicalTrials.gov in 2000. In addition, appended protocols are only provided with randomized controlled trails, which meant that our dataset was limited to RCTs and did not include single arm or non-randomized studies. Lastly, verbiage was often an issue when comparing endpoints in protocols and registries and for the purposes of our study, we accepted intent.

## Conclusions

Although recent regulations and enhanced procedures have improved the number and quality of clinical trial registrations, our study showed that there are still inconsistencies regarding the endpoint reporting within registries. The omissions of SEP reporting in clinical trial registries represent a critical limitation in the assessment of endpoint reporting. In addition, the presence of additional SEP in registries that are not in the clinical trial protocol may reflect the heterogeneity of protocol construction and suggests efforts to improve standardization and consistency within clinical trial protocols. Though further guidelines for the registration of clinical trials will help, greater efforts to provide a correct, easy to access and complete representation of planned endpoints are needed.

## Additional file

Additional file 1: Listing of all primary and secondary endpoints. (DOCX 29 kb)

## Abbreviations

CORRE: Comprehensive outcomes reporting in randomized evidence
FDA: U.S. Food and Drug Administration
ICMJE: International Committee of Medical Journal Editors
JCO: Journal of Clinical Oncology
NEJM: New England Journal of Medicine
NIH: National Institutes of Health
NPEP: Non-primary endpoint
PEP: Primary endpoint
PFS: Progression free survival
RCT: Randomized controlled trials
SAP: Statistical analysis plan
SEP: Secondary endpoint
TTP: Time to progression
WHO: World Health Organization
