# Supplementary material for: Discrepancies in endpoints between clinical trial protocols and clinical trial registration in randomized trials in oncology
Source: BMC Med Res Methodol. 2018 Dec 12;18:169. doi: 10.1186/s12874-018-0627-2 (PMC6292048; doi:10.1186/s12874-018-0627-2)
Supplement: Supplementary file 1 — Listing of all primary and secondary endpoints. (DOCX 29 kb) [file 12874_2018_627_MOESM1_ESM.docx]

**Appendix: Clinical Trial Primary and Secondary Endpoints from Protocol and Registry**

| Primary Endpoint- Protocol | Primary Endpoint- Registry | Discrepancy | Secondary Endpoints- Protocol | Secondary Endpoint- Registry | Discrepancy |
| --- | --- | --- | --- | --- | --- |
| Disease free interval | Disease free interval | No | QoL Postop length of hospital stay Sites of recurrence Surgical complication Peri-operative mortality | QoL Postop length of hospital stay Sites of recurrence Surgical complication Peri-operative mortality Changing from LAVH to TAH/BSO | Yes |
| DFS | DFS | No | RR OS  QoL  AE | RR OS  QoL  AE | No |
| PFS | PFS | No | ORR DoR OS Patient reported outcome changes in QoL/symptoms Safety | ORR DoR OS Patient reported outcome changes in QoL/symptoms | Yes |
| FFTF | FFTF | No | OS PFS Acute toxicity Late toxicity QoL CR Relapse rate Progression rate | None listed | Yes |
| OS | OS | No | RR DCR TTP QoL | ORR QoL PFS Safety Tissue samples | Yes |
| Fatigue severity | Fatigue severity | No | Improvements in health-related quality of life(EORTC) Energy conservation  Physical activity Nutrition Sleep hygiene Pain Distress | Symptom scores EORTC Stage of readiness (physical activity, nutrition, sleep hygiene, distress, pain control, energy conservation) | Yes |
| TTP | TTP | No | AE ORR Symptoms Plasma levels OS Molecular markers | ORR OS DoR | Yes |
| OS | OS | No | PFS Toxicity 50%decrease in PSA | PFS Toxicity 50%decrease in PSA | No |
| Local Control | Local Control | No | Larynx preservation Regional control rate Toxicity QoL DFS OS | Larynx preservation Regional control rate Toxicity QoL DFS OS | No |
| PFS | PFS | No | ORR TTP Osm DoR AE | ORR TTP Osm DoR Patient reported outcomes (AE) | No |
| ORR, Safety, PFS | ORR, Safety, PFS | No | PFS (random, all) Pharmacokinetics Pharmacodynamics Pharmacogenomics Metabolic Markers | PFS (random, all) Pharmacokinetics Pharmacodynamics | Yes |
| OS | OS | No | PFS Safety | None Listed | Yes |
| PFS | TTP | Yes | TTTF OS QoL RR DoR Secondary surgical resection rate Safety | OS QoL RR DoR Secondary surgical resection rate Safety | Yes |
| %pCR | %pCR | No | % clinical observation response % breast conserving surgery Safety TTTF % inhibition of intermediate and final biomarkers Correlation bewteen tumor gene expression at diagnosis and pathologic response | % clinical observation response % breast conserving surgery AE TTTF % of patients with tx failure % of inhibition of biomarkers Mutations at baseline | Yes |
| TTP | TTP | No | ORR TTF DoR Clinical benefit rate DoCBR OS AE | ORR TTF DoR Clinical benefit rate DoCBR OS | Yes |
| AUC for pain | AUC for pain | No | Potential risk factors for development of PIBP Identify potential clinical predictors of response or failure to naproxen Toxicity | Potential risk factors for development of PIBP Identify potential clinical predictors of response or failure to naproxen Toxicity | No |
| PFS | PFS | No | ORR OS DoR Toxicity | ORR OS DoR AE Gastrointestinal Perforation | Yes |
| OS | OS | No | PFS ORR 1 yr survival DoR AE Patient reported outcomes | PFS ORR 1 yr survival DoR Patient reported outcomes | Y |
| OS | OS | No | ORR DoR EFS Safety DFS 4 month EFS | ORR DoR EFS Safety DFS 4 month EFS | No |
| Impact in attitude | Impact in attitude | No | Knowledge Self-efficacy Receptivity Likelihood of participation | Knowledge Self-efficacy Receptivity Likelihood of participation | No |
| TTP | TTP | No | RR (CRR, ORR) AE OS | RR (CRR, ORR) AE OS Rate of disease stabilization (VEGF) | Yes |
| TTP | TTP | No | RR (CR, PR, MR) OS AE | RR (CR, PR, MR) OS AE | No |
| OS | OS | No | DFS OS Toxicity | OS DFS Late sequelae Nephrotoxicity | No |
| OS | OS | No | CRR AE | CRR | Yes |
| Change in Ki67 | Change in Ki67 | No | Effect of metformin by ER status Effect by HOMA Effect by BMI Change in Ki67 in ductal epithelial neoplasia Regulation of mitochondrial function Tissue biomarker analysis | Effect of metformin on Ki-67 by HOMA Effect on circulating antibodies Antiproliferative activity on hyperplastic/dysplastic/malignant breast tissue | Yes |
| EFS OS | EFS | Yes | RR Neuropsychological and neuroendocrine sequelae Prognostic factors | OS Tumor response rates Toxic effect QoL Predictive factors Factors contributing to neuropsychological and endocrine status | Yes |
| BOR | BOR | No | OS TTR PFS Safety Correlative MAPK, AKT, Ki67 and EGFR dependent signaling Markers and gene expression in CTC | OS TTR PFS Safety | Yes |
| CBR | CBR | No | RR TTP AE OS Serum DNA alterations | AE OS CR PR | Yes |
| Medical knowledge | Medical knowledge | No | Decisional conflict Regret Treatment preference Final treatment choice & rationale | Decisional conflict Regret Treatment preference Final treatment choice & rationale Feeling Informed | Yes |
| PFS | PFS | No | Response OS Toxicity PFS HDM PFS allo | Response OS Toxicity PFS HDM PFS allo | No |
| OS | OS | No | PFS AE RR QoL Health resource and economic evaluation PFS rate on erlotinib at 9 weeks RR 1st line PFS 1st line | PFS AE RR QoL Health resource and economic evaluation PFS rate on erlotinib at 9 weeks RR 1st line PFS 1st line | No |
| OS | OS | No | AE PFS Resection assessment | AE PFS | Yes |
| OS | OS | No | PFS TR DoCR DoR DoSD QoL TTP Change in performance status Lung cancer symptom response AE | PFS TR DoCR DoR DoSD QoL TTP Lung cancer symptom response | Yes |
| EFS | EFS | No | PFS OS Pattern QoL AE TTP Biomarkers Ototoxicity Endocrine late effects | OS Pattern QoL Hearing loss Endocrine deficiencies Toxicity | Yes |
| Severity of delayed nausea | Severity of delayed nausea | No | QoL (reducing interference with functioning due to CINV) X 3 | QoL | No |
| PFS | PFS | No | OS AE Predictive value of EGFR mutation & expression (RR, PFS, OS) Symptoms evaluation | OS Toxicity QoL | Yes |
| CCyR | CCyR | No | Major Molecular Response Duration of CCyR Complete Hematologic Response Population Pharmacokinetics Time to transformation to Ap/BP Safety | Major Molecular Response Duration of CCyR Complete Hematologic Response Population Pharmacokinetics Time to transformation to Ap/BP | Yes |
| OS PFS | OS | Yes | RR DoR Liver resection rate AE Wound healing | RR DoR Liver resection rate Wound healing Best % chang in tumor size | Yes |
| PFS | PFS | No | RR DOR OS QoL Time to worsening of QoL Symptom worsening | RR DOR OS QoL Time to worsening of QoL Symptom worsening % Change in tumor size | Yes |
| OS | OS | No | PFS RR QoL AE Pharmacokinetics Immunogenicity of aflibercept | PFS RR QoL | Yes |
| pain control | pain control | No | Brief Pain Inventory QoL Karnofsky Opioid use BPI pain QoL >6 days | Brief Pain Inventory QoL Karnofsky Opioid use | Yes |
| Fitgue improvement | Fitgue improvement | No | Prevalence of deficiency Effect of carnitine on pain at 4 and 8 week Performance at 4 and 8 weeks Brief fatigue Brief Pain Inventory | Prevalence of deficiency Effect of carnitine on pain at 4 and 8 week Performance at 4 and 8 weeks Toxicity Brief Pain Inventory | No |
| OS | OS | No | PFS AE ORR DOR Pharmacokinetics/Pharmacodynamics QoL Best response TTP CA-125 response Cardiac QT Beta-tubulin FOSI Symptoms Composite ORR-CA125 | PFS AE DOR Pharmacokinetics/Pharmacodynamics Best response TTP Cmin | Yes |
| LR | LR | No | Acute Toxicity Chronic Toxicity Resection rate QoL OS DFS LRFS TTDR Surgical Complications | Acute Toxicity Chronic Toxicity Resection rate QoL OS | Yes |
| EFS | EFS | No | OS EWS (Ewing's Sarcoma) prognostic EWS (Ewing's Sarcoma) for minimal disease assessment Tumor biology to clinical features | None listed | Yes |
| Menopausal Symptoms | Menopausal Symptoms | No | Urinary symptoms Sexuality Body image and self image Psychological distress Generic health-related quality of life (HRQL) | Urinary symptoms Sexuality Body image and self image Psychological distress Generic health-related quality of life (HRQL) Vasomotor Symptoms | No |
| OS | OS | No | DFS AE IF-a-2b QoL Prognostic factors | DFS AE Time series of markers Role/Mechanism of IF-a-2b in patient regimen QoL Prognostic factors | Yes |
| PFS | PFS | No | OS Local Control Time to Distant Metastasis Toxicity MDR1 DFS MDR1 Myelosuppression Menopause Induction | OS Local Control Time to Distant Metastasis Toxicity MDR1 DFS MDR1 Myelosuppression Menopause Induction Neutropenia Peripheral Neuropathy | Yes |
| General fatigue | General fatigue | No | Anxiety Depression QoL Mental fatigue | Anxiety Depression QoL Mental fatigue | No |
| TTP | TTP | No | RR DoR Toxicity  OS | RR DoR Toxicity  OS | No |
| PFS | PFS | No | OS TTP ORR DoR CA125 response Disease related symptoms  QoL AE | OS TTP ORR TFST/TSST/TDT QoL AE Pharmacokinetics PFS in BRCA Time to 2nd progression CA125 | Yes |
| DFS | DFS | No | Metastasis free survival Late toxicity 1/2 yrs Bladder capacity QoL | Metastasis free survival Late toxicity 1/2 yrs Bladder capacity QoL | No |
| ablation rate | ablation rate | No | Qol Cost Hypothyroid Symptoms Toxicity  QoL | Qol Cost Hypothyroid Symptoms Toxicity  QoL Long Term Complications | No |
| OS, QoL | OS, QoL | No | Path response PFS N of resection AE Cost QoL | Path response PFS N of resection AE Cost QoL | No |
| PFS | PFS | No | OS TTP RR TTR DOR TTT AE QoL | OS TTP RR TTR  DOR TTT AE QoL | No |
| TTP | TTP | No | CR  OS PFS Feasibility | CR  OS PFS Feasibility | No |
| Post transplant PFS | Post transplant PFS | No | OS RR TTR  DoR DoCR TTP | OS RR | Yes |
| OS | OS | No | QoL TTP ORR DoR Number of disease-free patients Impact of mitotane drug levels on outcomes Impact of 2nd line treatment | QoL TTP ORR DoR Number of disease-free patients Impact of mitotane drug levels on outcomes Impact of 2nd line treatment Pharmacokinetics or mitotane | Yes |
| All cause mortality | All cause mortality | No | Cancer mortality  PFS QoL Disease recurrence rate Bone metastasis 30 day surgical complication | Cancer mortality  PFS QoL Disease recurrence rate Bone metastasis 30 day surgical complication Symptoms Need for cancer tx RR | Yes |
| CR, PFS | CR, PFS | No | OS | OS TTF Side effects | Yes |
| TTP | TTP | No | CR OS AE Prognostic significance of ER and HER2 Estrogen parameters (estrogen levels) Anastrozole levels Estradiol levels | CR OS AE Prognostic significance of ER and HER2 Estrogen parameters (estrogen levels) Anastrozole levels Estradiol levels | No |
| OS | OS | No | PFS Time to skeletal QoL PSA progression AE  Pain palliation CTC rate ECG Changes Pharmacokinetics | PFS Time to skeletal QoL PSA progression AE  Pain palliation CTC rate Soft tissue objective response | Yes |
| PFS OS Safety | PFS | Yes | PFS  ORR DoR CR TTF Time to symptom progression Resource expenditure | ORR DoR  CR Time to symptom progression OS Serum concentration trastuzumab Plasma concentration free emtansine | Yes |
| FFTF | FFTF | No | PFS  OS | QoL Prognostic significance of FDG-PET | Yes |
| PFS | PFS | No | OS PFS  ORR DOR Braf Mutation prediction | OS PFS  ORR DOR Braf Mutation prediction  Non-melanoma condyloma | Yes |
| OS | OS | No | PFS ORR DoCR DoR TDS AE Pharmacodynamics Genetics | PFS ORR DoCR DoR TDS | Yes |
| OS | OS | No | PFS OS in PIGF analysis subset ORR DOR Pharmacokinetics AMG Pharmacokinetics carboplatin AE Change in labs | PFS OS in PIGF analysis subset ORR DOR Pharmacokinetics AMG Pharmacokinetics carboplatin AE | Yes |
| OS | OS | No | Neutrophil engraftment Primary Graft failure Secondary Graft failure Platelet engraftment Acute GVHD Chronic GVHD Immunosuppressive free interval Relapse Infection Immune reconstitution Patient QOL Donor QOL Donor recovery (3 parameters) AE | Neutrophil engraftment Primary Graft failure Secondary Graft failure Platelet engraftment Acute GVHD Chronic GVHD Immunosuppressive free interval Relapse Infection Immune reconstitution Patient QOL Donor QOL Donor recovery (3 parameters) AE | No |
| OS | OS | No | Time to hormonal resistance QOL Serum cholesterol HDL LDL Bone density Duration of treatment Nontreatment intervals Time to testosterone recovery Time to recover of potency CAG repeats on the AR in cfDNA | Time to hormonal resistance QOL Serum cholesterol HDL LDL Duration of treatment Nontreatment intervals Time to testosterone recovery Time to recover of potency | Yes |
| PFS in V600+ | PFS in V600+ | No | ORR ORR V600K PFS V600K DOR PFS No prior chemo  PFS 1 prior chemo  AE | PFS PFS in V600+ no chemo hx PFS in v600 +chemo hx OS OS in BRAF+ ORR ORR in BRAF +  RR in BRF+ RR  OR following crossover to trametinib DoR DoR following crossover PFS following crossover | Yes |
| Remnant ablation of 6-9 | Remnant ablation of 6-9 | No | QoL Loco-regional performance Distant metastasis Survival Secondary primary malignancy Length of isolation Social factors Economic factors | QoL Loco-regional performance Distant metastasis Survival Secondary primary malignancy | Yes |

ABBREVIATIONS: DFS= Disease free survival, PFS= Progression free survival, FFTF= Freedom from treatment failure, OS= Overall survival, TTP= Time to progression, ORR= Objective response rate, %pCR= Percentage pathologic complete response, AUC= Area under the curve, EFS= Event free survival, CCyR= Complete cytogenetic response, LR= Local recurrence rate, QoL= Quality of Life, CR= Complete response, LAVH= Laparoscopically assisted vaginal hysterectomy, TAH/BSO= Total abdominal hysterectomy/Bilateral salpingo-oopherectomy, RR= Response rate, AE= adverse events, DoR= duration of response, DCR= Duration of complete response, DoCBR= Duration of clinical benefit, PR= Partial response, MR= Mixed response, TTR= Time to response, DoSD= Duration of stable disease, LRFS= local recurrence free survival, TTDR= Time to Disease Recurrence, TFST= Time to first subsequent therapy, TSST= Time to second subsequent therapy, TDT= Time to treatment discontinuation, TTT= Time to next treatment
